# Simultaneous Determination of Residual Contamination of Eight Antineoplastic on Surfaces by HILIC Chromatography Coupled to High‐Resolution Spectrometry

**DOI:** 10.1002/ansa.70004

**Published:** 2025-02-27

**Authors:** Zribi Kaouther, Sarra Berriri, Danielle Libong, Audrey Solgadi, Fathi Safta, Laetitia Minh Mai Lê, Eric Caudron

**Affiliations:** ^1^ LR 12ES09 Faculty of Pharmacy of Monastir University of Monastir Monastir Tunisia; ^2^ Laboratory of Hygiene Hedi Chaker University Hospital Sfax Tunisia; ^3^ Pharmacy Department Farhat Hached University Hospital Center Sousse Tunisia; ^4^ Lipids, Analytical and Biological Systems University of Paris‐Saclay Orsay France; ^5^ UMS‐IPSIT SAMM Facility Inserm CNRS Engineering and Platforms for Therapeutic Innovation UFR Pharmacy University of Paris‐Saclay Orsay France; ^6^ Pharmacy Department European Georges Pompidou Hospital APHP Centre—University of Paris Cité Paris France

**Keywords:** antineoplastic drugs, healthcare worker safety, HILIC separation, mass spectrometry

## Abstract

Residual contamination by intravenous antineoplastic drugs on hospital surfaces remains a critical concern, as highlighted by numerous studies. This study presents a novel, rapid and highly sensitive analytical method for quantifying a wide range of antineoplastic drugs and detecting other potentially harmful molecules on wiped surfaces. Utilizing hydrophilic interaction liquid chromatography (HILIC) coupled with high‐resolution spectrometry, the method combines the quantification of eight commonly used antineoplastic drugs: 5‐fluorouracil, ifosfamide, cyclophosphamide, gemcitabine, doxorubicin, methotrexate, epirubicin and irinotecan, with the identification of unknown compounds offering a comprehensive solution for monitoring hospital surface contamination. While HILIC‐MS/MS has been extensively applied in various matrices, its use for surface contamination monitoring in healthcare settings has been relatively underexplored. Chromatographic separation was achieved using gradient elution on an HILIC ZORBAX 120 column (150 mm × 2.1 mm, 4 µm), enabling rapid analysis within 8 min. The method demonstrated exceptional sensitivity, achieving limits of quantification below 0.04 ng/cm^2^ for all targeted molecules. Applied to 28 surfaces in the day hospital of a medical oncology unit at a French hospital, the method revealed contamination on 22 surfaces with at least one antineoplastic drug. Additionally, unknown molecules, including a compound associated with cleaning detergents, were detected, highlighting the complexity of hospital surface contamination underscoring the ongoing risks faced by healthcare workers and patients. This innovative approach represents a significant advancement in analytical chemistry and hospital hygiene monitoring, providing a faster, more efficient and versatile alternative to traditional techniques, as it allows 5‐FU quantification within the same run time with other molecules. By addressing critical gaps in current methodologies, this study offers valuable insights into occupational safety and supports efforts to reduce exposure risks for healthcare workers and patients. Further research is needed to identify the unknown molecules detected and fully assess their potential risks.

## 1 Introduction

According to the latest report from the World Health Organization (WHO) and the International Agency for Research on Cancer (IARC), the global cancer burden reached 19.3 million new cases in 2020, with cancer accounting for 10.0 million deaths in 2020 [1]. This surge in cancer cases has driven a corresponding increase in the use of antineoplastic drugs, classified as hazardous by the National Institute for Occupational Safety and Health (NIOSH) due to their carcinogenic, mutagenic and teratogenic properties [2]. Consequently, healthcare workers are at heightened risk of occupational exposure to these drugs through direct contact, inhalation or ingestion of contaminated surfaces and equipment.

Numerous studies have documented residual contamination of hospital surfaces with cytotoxic drugs, particularly in chemotherapy preparation areas, patient rooms and treatment zones. These drugs can persist long after administration, underscoring the need for robust monitoring methods and stringent cleaning protocols to mitigate exposure risks. Even trace amounts of these hazardous substances pose significant health threats to healthcare workers, emphasizing the critical importance of environmental monitoring in healthcare settings [3, 4, 5, 6, 7, 8, 9].

Despite growing concerns, few studies have systematically screened for other potentially present compounds, such as degradation products of cytotoxic drugs, premedication drugs associated with anticancer protocols or cleaning agents used in healthcare environments. This gap highlights the need for more comprehensive monitoring methods capable of addressing the complexity of hospital contamination [3, 4, 5, 6, 7, 8, 9].

Wipe sampling is the most commonly employed method for assessing surface contamination by antineoplastic drugs in hospitals. Traditional analytical techniques, such as liquid chromatography–mass spectrometry (LC‐MS/MS) [3, 8, 9], atomic absorption spectrometry [4] and reversed‐phase liquid chromatography (RPLC) [5], have been widely employed for detecting these compounds. However, these methods often target a limited range of drugs, require lengthy analysis times or struggle to effectively retain and separate small, polar compounds like 5‐fluorouracil (5‐FU) on conventional reversed‐phase columns. This often necessitates multiple analytical methods to detect all relevant analytes. This challenge highlights the need for more comprehensive and efficient analytical approaches.

Hydrophilic interaction liquid chromatography (HILIC) coupled with mass spectrometry (HILIC‐MS) offers a promising alternative for analysing polar and hydrophilic compounds. HILIC provides enhanced retention and separation, making it particularly suitable for detecting and quantifying antineoplastic drugs in complex environmental samples. While HILIC has been widely used for various applications, its specific use in the analysis of antineoplastic drugs has been explored by several studies. Ares and Bernal reviewed the application of HILIC in drug analysis, emphasizing its potential for detecting polar and small‐molecule compounds, which is critical for environmental monitoring in healthcare settings [10, 11].

Building on the foundational work of Ares and Bernal [11], this study extends the application of HILIC‐MS/MS to a broader range of compounds, including degradation products and premedication drugs. While previous studies have primarily focused on biological or environmental matrices, significant gaps remain in its application for hospital surface contamination monitoring. For instance, Sessink et al. [12, 13] concentrated on air samples or biological fluids, often quantifying a limited number of drugs. Contamination of hospital surfaces, however, presents unique challenges that require specialized approaches.

Recent advancements, such as the studies by Dugheri et al. [14, 15], have demonstrated the applicability of HILIC‐MS/MS for detecting platinum‐based drugs on hospital surfaces, expanding its role in environmental health and safety research. Dugheri et al. [14] focused on characterizing platinum‐based drugs, while their 2022 study [15] broadened the scope to include a wider range of antineoplastic drugs using zwitterionic HILIC methods. However, challenges persist, including method development and mitigating matrix effects. Moreover, the simultaneous quantification of a broader spectrum of antineoplastic drugs at ultra‐trace levels, along with the identification of non‐targeted compounds, remains an unmet need.

Building on these studies, this research introduces a novel, validated HILIC‐MS/MS method designed to address these limitations. The developed method was designed to address the challenge of simultaneously quantifying a diverse range of antineoplastic drugs with varying physicochemical properties. The selection of HILIC was informed by its unique ability to retain polar and hydrophilic compounds effectively. Unlike reverse‐phase chromatography, which struggles with polar analytes, HILIC allows for better separation due to the hydrophilic stationary phase and the high organic content of the mobile phase. This choice also enhances sensitivity in electrospray ionization (ESI), particularly in positive mode, which is crucial for detecting low‐abundance compounds on contaminated surfaces. The method enables rapid analysis with high sensitivity, achieving limits of quantification below 0.04 ng/cm^2^, and incorporates high‐resolution Orbitrap spectroscopy for identifying non‐targeted compounds. These compounds include degradation products, premedication drugs and cleaning agent residues, all of which may contribute to occupational exposure risks. By detecting antineoplastic contaminants at trace levels below the United States Pharmacopeia (USP) contamination limit of 1 ng/cm^2^ [2, 16], this study provides a comprehensive approach to environmental monitoring.

The selection of antineoplastic drugs for this study was guided by their frequent use in hospital settings. The eight targeted drugs—doxorubicin, epirubicin, cyclophosphamide, methotrexate, irinotecan, gemcitabine, ifosfamide and 5‐FU—accounted for over 50% of all cytotoxic drug preparations dispensed in a day hospital in 2023. This focus ensures the method's relevance to real‐world clinical practices while addressing critical gaps in current monitoring methodologies.

By bridging targeted and non‐targeted analyses, this study represents a significant advancement in hospital hygiene monitoring. It not only enhances our understanding of surface contamination but also supports the development of improved safety protocols and regulatory standards. These findings are pivotal for reducing occupational exposure risks and safeguarding healthcare workers from the harmful effects of antineoplastic drugs.

## 2 Materials and Methods

### 2.1 Chemicals and Reagents

Drug standards were prepared using commercially available drugs from various manufacturers. Specifically,

- 5‐FU (500 mg/10 mL) and doxorubicin (50 mg/25 mL) were obtained from Accord Healthcare;
- cyclophosphamide (1000 mg) and ifosfamide (2000 mg) powders for injection were sourced from Baxter, reconstituted with 50 mL of 0.9% sodium chloride (Fresenius) and 50 mL of water for injection (Proamp), respectively;
- epirubicin (200 mg/100 mL), methotrexate (50 mg/2 mL), irinotecan (500 mg/25 mL) and gemcitabine (2000 mg/50 mL) were provided by Mylan.

LC‐MS solvents, including acetonitrile (ACN), LC‐MS water and formic acid, were purchased from Sigma Aldrich. The chemical structures, physicochemical properties, molecular formulas and average masses of the eight targeted molecules are summarized in Table 1.

**TABLE 1:** Physicochemicals properties of the molecules.

Molecule and molecular formula	Average mass (uma)	p*K*a	log*P*	Structure
5‐Fluorouracil C_4_H_3_FN_2_O_2_	130.777	p*K*a_1_ = 7.5 p*K*a_2_ = 9.0	−0.89	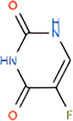
Cyclophosphamide C_7_H_15_Cl_2_N_2_O_2_P	261.086	p*K*a_1_ = 2.3 p*K*a_2_ = 11.1	0.63	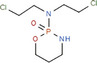
Ifosfamide C_7_H_15_Cl_2_N_2_O_2_P	261.086	p*K*a_1_ = 2.5 p*K*a_2_ = 9.1	0.86	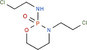
Doxorubicin C_27_H_29_NO_11_	543.519	p*K*a_1_ = 7.34 p*K*a_2_ = 8.46 p*K*a_3_ = 9.46	1.27	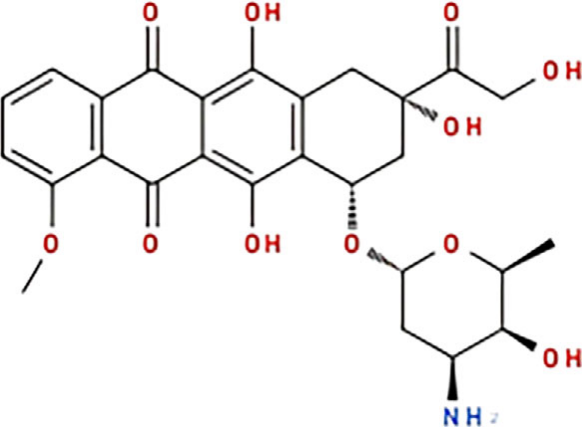
Epirubicin C_27_H_29_NO_11_	543.519	8.94	1.41	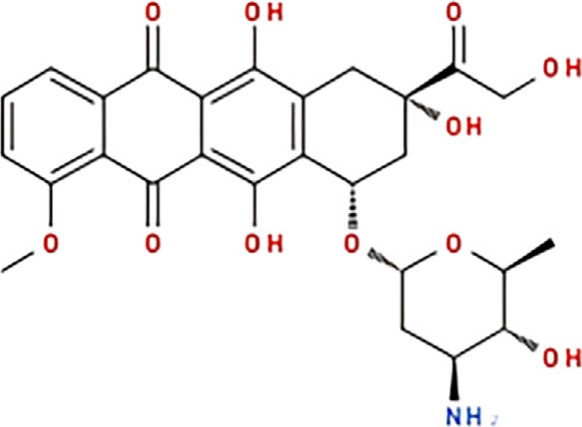
Methotrexate C_20_H_22_N_8_O_5_	454.439	p*K*a_1_ = 2.9 p*K*a_2_ = 4.6 p*K*a_3_ = 6.6	−1.85	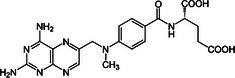
Irinotecan C_33_H_38_N_4_O_6_	586.678	p*K*a _1_ = 9.47 p*K*a_2_ = 11.7	3.2	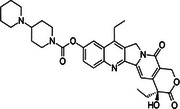
Gemcitabine C_9_H_11_F_2_N_3_O_4_	263.198	3.6	−1.4	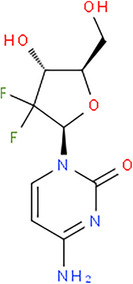

### 2.2 Instrumentation and Chromatographic Conditions

Analyses were conducted using an Ultimate 3000 Dionex HPLC system (Thermo Fisher Scientific, Milan, Italy) equipped with a quaternary RS pump, RS autosampler and solvent platform. Separation was achieved using a Zorbax HILIC column (150 mm × 2.1 mm, 4 µm, Agilent). Two other columns have been tested during method development: HILIC Zorbax Plus (50 mm × 2.1 mm, 3.5 µm) and HILIC Zorbax NH2 (150 mm × 2.0 mm, 3 µm). Compound detection was performed with an LTQ‐Orbitrap Velos Pro (Thermo Fisher Scientific) equipped with an ESI source for targeted quantitative analysis and untargeted screening.

Chromatographic conditions included ambient column temperature, mobile phase flow rate set at 0.35 mL/min with a linear gradient (Data S1) and an injection volume of 10 µL.

Following separation, the effluent was directed to the ESI source using nitrogen as sheath and auxiliary gas. The main tuning parameters for the ESI source were as follow: a capillary voltage of 3000 V, ion energy fixed at 0.5 eV, source temperature set to 150°C, desolvation temperature maintained at 350°C and desolvation flow rate fixed at 450 L/h.

Initially, the negative ion mode (ESI−) was applied for 1.45 min to detect 5‐FU, followed by switching to the positive ion mode (ESI+) for the detection of the other compounds in the same run. 5‐FU was detected using full scan mode with high resolution within the mass range of 129.000–129.016 uma. The other compounds were detected using MS/MS mode. The instrument method for the Orbitrap Velos Pro is summarized in Data S2.

### 2.3 Sample Preparation

#### 2.3.1 Mobile Phase Solutions

HILIC was performed using a gradient of elution method. With eluents of water and ACN, each containing 0.1% formic acid. A linear gradient of ACN (85%–95%) was applied over duration of 7.5 min, as outlined in Data S1, optimizing the HILIC conditions.

#### 2.3.2 Cytotoxic Stock Solutions, Calibration and Validation Standards

Stock solutions, calibration standards and validation standards were prepared in accordance with cytotoxic drugs handling regulations, adhering to strict safety protocols using closed‐system drug‐transfer devices and laminar airflow equipment to minimize operator exposure to hazardous compounds during preparation.

Stock solutions of each drug were extemporaneously prepared with concentrations of 200,000, 4000, 400 and 40 ng/mL. These solutions were used to prepare five calibration standards and three validation standards ranging from 0.1 to 200 ng/mL, ensuring a comprehensive range for accurate quantification and validation of the analytical method.

### 2.4 Method Validation

Validation was performed according to harmonized strategies for the validation of quantitative analytical procedures [17]. The validation process was carried out over 3 successive days. Each day, three replicates of calibration standards were analysed, following by the analysis of three replicates of validation standards to establish model validation. Intra‐day and inter‐day precision were calculated to access the consistency of results within and between days, respectively. Trueness was estimated by comparing the calculated concentration with the true concentration for each point. Precision (intra‐day) and intermediate precision (inter‐day) were estimated using the relative standard deviation (RSD %).

The limit of quantification (LOQ) was determined based on a signal‐to‐noise ratio greater than 10. This criterion ensures that the signal from the analyte is distinguishable from background noise with a sufficient level of confidence.

### 2.5 Application to Surface Contamination Monitoring

Surface wiping was performed in accordance with the method outlined by Nussbaumer et al. [18]. Especially, 1 cm × 1 cm filter paper (Protein Saver 903 card) from Whatman (Dassel, Germany) was utilized. The filter paper was wetted with 100 µL of a mixture of ACN with 0.1% formic acid and water solution with 0.1% formic acid (80/20, v/v). Desorption of the analytes from the filter paper was carried out in a polypropylene tube using 1 mL of initial mobile phase of the gradient described in Data S1. Subsequently, ultrasonication was performed for duration of 20 min to ensure efficient desorption of the analytes from the filter paper.

A preliminary study of surface contamination was performed in the day hospital of a medical oncology unit at a French hospital. The pharmacy department was responsible for preparing chemotherapy, and the preparation medications were then delivered to the oncology department for patient administration.

A total of 28 wipe samples were collected from various potentially contaminated areas within the unit, each measuring 10 cm × 10 cm. These samples comprised six from patient's rooms (including arm‐rest, remote control, pump, floor under the pump, perfusion support and table), six from the patient's toilets (including the seat, floor, toilet flush, door handle, toilet cover and tap) and 16 from the nursing station (including the floor, various points on the bench, storage back and the keyboard of the nursing trolley). The surfaces at the nursing station were wiped during a typical working day, without disrupting the nurses’ activities. The patient's room was wiped after daily cleaning room protocol.

The screening of untargeted molecules was conducted across the 28 samples, where exact mass of the ions found on the samples were compared to those described in the literature and to molecules that could potentially be present on the surfaces (such as other drugs or detergent components). This included the possibility of untargeted antineoplastic compounds or degradation products being present. Identification of these untargeted molecules was carried out chromatographic peak exhibiting signal‐to‐noise ratio greater than 10. This identification process involved comparing the exact mass of the untargeted molecule with the masses of other antineoplastics, their degradation products, other drugs co‐administrated within chemotherapy protocol and potentially presents detergent components.

## 3 Results and Discussion

### 3.1 Method Development

During method optimization, three different HILIC columns were tested: the HILIC Zorbax Plus (50 mm × 2.1 mm, 3.5 µm), HILIC Zorbax NH2 (150 mm × 2.0 mm, 3 µm) and HILIC Zorbax 120 (150 mm × 2.1 mm, 4 µm). For the first two columns, irinotecan was the first compound to elute, with retention times of 1.12 and 1.63 min, respectively. However, in our method, using the third column, irinotecan eluted last at 6.72 min.

This variation in elution order highlights the importance of column selection in HILIC chromatography. The elution order of the different molecules was not the same. 5‐FU was the first to elute only on HILIC Zorbax 120, suggesting that the polarity is not the only physicochemical parameter to be considered. In fact, HILIC separation integrates reversed and normal phase chromatography, as well as adsorption and ion exchange mechanisms. Selectivity differs then depending on the interaction between the mobile phase, stationary phase and the analyte. In this study, the three HILIC columns tested differed in their stationary phase chemistries and particle sizes, which significantly influenced their retention behaviour. The HILIC Zorbax Plus column (3.5 µm particle size) features a standard silica stationary phase, promoting strong hydrophilic interactions. The HILIC Zorbax NH2 column (3 µm particle size) is amino‐based, introducing additional ion exchange interactions due to the amino groups on the stationary phase. In contrast, the HILIC Zorbax 120 column (4 µm particle size) utilizes a silica‐based stationary phase with a different porosity and surface chemistry, potentially altering the balance of hydrophilic partitioning and adsorption effects. These differences in stationary phase composition and particle size influence the strength and type of interactions between the analytes, the mobile phase and the stationary phase.

Silica‐based HILIC Zorbax 120 (150 mm × 2.1 mm, 4 µm) from Agilent was ultimately selected for its ability to elute 5‐FU first, which is a polar molecule, enabling its detection in ESI− followed by the detection of other molecules in ESI+. This unique behaviour suggests the possibility of setting up an initial detection window in ESI− mode for 5‐FU, followed by a transition to ESI+ mode for the analysis of other compounds.

Various flow rates and proportions of the ACN/water mixture were tested during optimization. Several mobile phases with organic solvent percentages from 5% to 25% were evaluated. The selected mobile phase, used as the initial phase of the gradient, provided sufficient resolution between 5‐FU and cyclophosphamide, allowing for a smooth transition from ESI− to ESI+. As for the flow rate, an initial rate of 0.3 mL/min was used, but it was later increased to 0.35 mL/min to reduce the analysis time without compromising the resolution.

**FIGURE 1:**
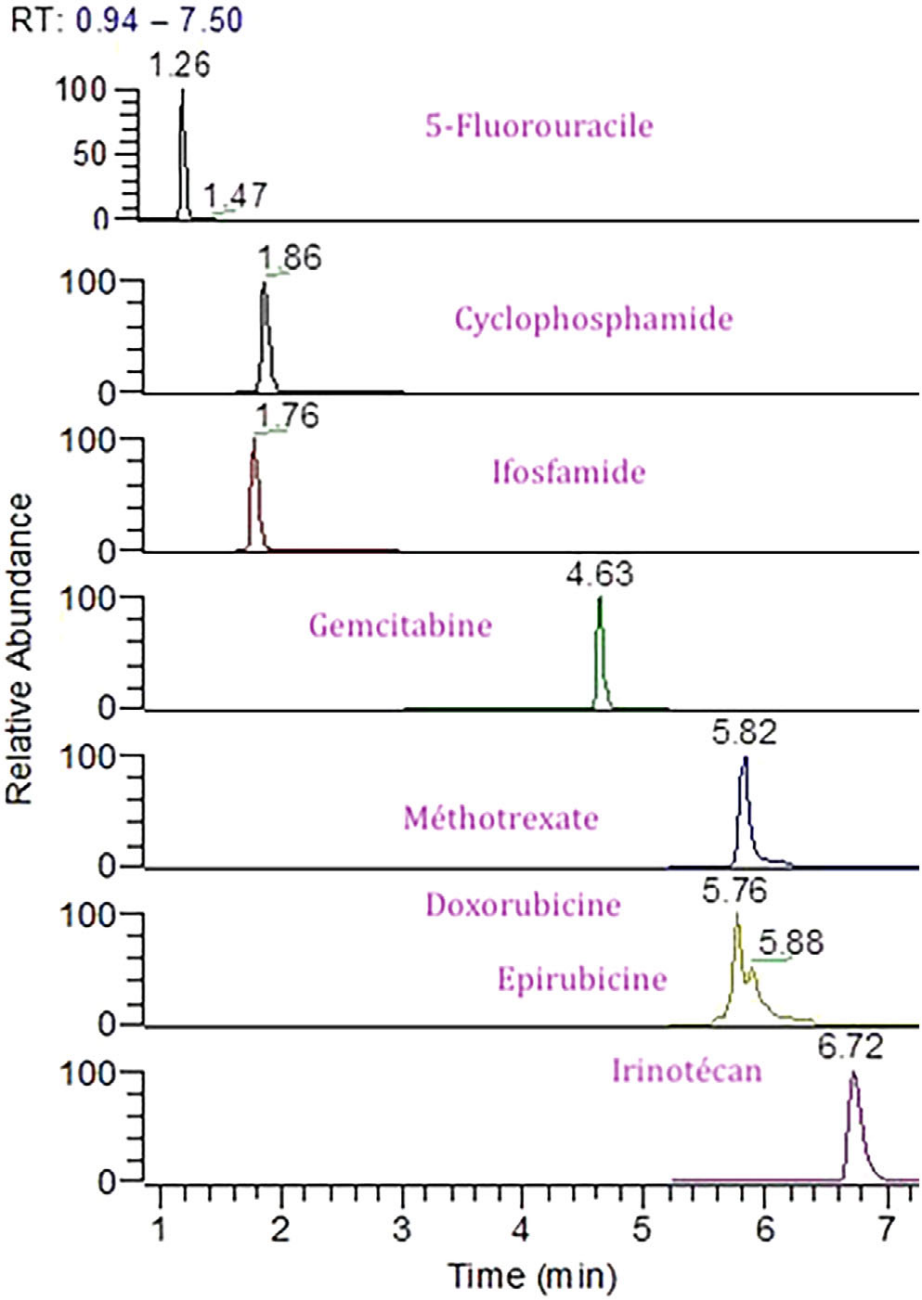
Chromatographic separation of drugs detected in negative mode (5‐flurouracil) and in positive mode (other drugs).

We chose the chromatographic parameters that yielded the best results across different fixed challenges, including achieving a single analytical run for the analysis of 5‐FU with other antineoplastic drugs, maintaining an acceptable lower LOQ and ensuring a short run time. This approach allowed for an increased number of samples to be analysed enhancing the representativeness of the results and improving the accuracy of the contamination map. The chromatographic separation is depicted in Figure 1.

The optimization of the flow rate and ACN content was conducted systematically to ensure reproducibility and peak resolution while minimizing analysis time. A flow rate of 0.35 mL/min was selected as optimal, providing sufficient resolution for all analytes without compromising the column's performance or system pressure limits. Gradient elution was employed to achieve optimal separation, with initial conditions involving a high ACN content to facilitate retention of polar compounds. Gradual decreases in ACN content allowed for the elution of less polar analytes.

The HPLC run provided good separation for analytes detected in both ESI− and ESI+, with the exception for methotrexate, epirubicin and doxorubicin. Methotrexate exhibited a specific transition, while doxorubicin and epirubicin, due to their similar structure, shared the same transitions with a similar retention time. However, the two peaks were adequately separated at their apexes, allowing quantification to be performed based on peak height. Cyclophosphamide and ifosfamide, being isomers with identical molecular masses, were co‐eluted.

Despite the co‐elution, their structural differences resulted in distinct fragmentation patterns, enabling simultaneous quantification. Although the parent ion mass is identical for both molecules, their fragment ions differ as demonstrated by Dal Bello et al. [5]. The retained products ions were *m/z* 182.0 for ifosfamide and *m/z* 140.0 for cyclophosphamide. The specificity of the transition was confirmed through individual injections of cyclophosphamide and ifosfamide, which showed no signal overlap. This test confirmed the specificity of the transition.

**FIGURE 2:**
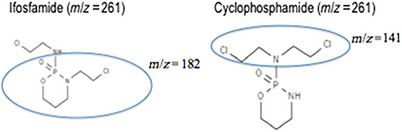
Specific fragments of ifosfamide and cyclophosphamide for selective detection.

Liu et al. [19] further corroborated these findings, publishing detailed fragmentation pathways for cyclophosphamide and ifosfamide, illustrating that the transitions used in our analysis are indeed specific. Figure 2 illustrates possible pathways for the formation of the fragment *m/z* 140 from protonated cyclophosphamide.

The elution order observed in the chromatographic separation was as follows: 5‐FU, ifosfamide/cyclophosphamide, gemcitabine, doxorubicin, methotrexate, epirubicin/doxorubicin and finally irinotecan.

Notably, the elution order does not strictly follow molecular polarity. For instance, while irinotecan is the least polar molecule, it does not elute first. Elution order is influenced by multiple factors, including polarity, molecular size, shape and interactions with the stationary phase [10]. Thus, the observed elution sequence reflects a combination of these factors rather than polarity alone as described previously.

5‐FU typically exhibits poor retention on reverse‐phase column due to its small size and high polarity. Its detection in ESI− by ESI further supports the need for a specific chromatographic run for this widely used cytotoxic drug. However, when using an HILIC phase, 5‐FU can be adequately retained, allowing its quantitative and specific determination. Usually, in HILIC separations, less polar molecules are typically eluted first [20]. However, in our study, 5‐FU was the first eluted despite being a polar molecule. This unexpected elution order highlights the complexity of retention mechanisms in HILIC. In HILIC mode, stationary phases typically used for normal‐phase liquid chromatography are used with RPLC mobile phase. This allows for the analysis of both polar and ionic analytes, resembling aspects of ion chromatography [10]. Thus, the unique combination of stationary and mobile phase in HILIC chromatography contributes to the complex retention behaviour observed, including the unexpected elution order of 5‐FU.

To improve the LOQ for 5‐FU, Tuerk et al. proposed a single method using HPLC‐APCI‐MS/MS for 5‐FU and a multiple method using HPLC‐ESI‐MS/MS for the determination of other molecules [21]. This strategy aimed to optimize the detection and quantification of 5‐FU while ensuring accurate analysis of the other compounds. By employing different detection modes within the same chromatographic run, our approach allows for comprehensive and efficient analysis of all target molecules.

Hao et al. [22] also proposed a method to determine residual contamination of 5‐FU, cyclophosphamide and gemcitabine. Their approach involved using two different methods: the first one employed ESI− to determine 5‐FU, while the second one utilized ESI+ to detect residual contaminations with cyclophosphamide and gemcitabine. This dual‐method strategy enabled the specific and sensitive determination of each compound, tailored to their respective detection requirements. Dugheri et al. also used LC‐MS/MS, employing two LC columns tailored to the properties of the cytotoxic drugs under investigation. For low‐polar and hydrophobic drugs, they used Atlantis T3 columns, which are silica‐based, reversed‐phase C18 column. This column was used for the analysis of compounds such as cyclophosphamide, ifosfamide, doxorubicin, methotrexate, epirubicin and irinotecan. To address the poor retention and separation difficulties encountered with polar and hydrophilic compounds like 5‐FU and gemcitabine on reversed‐phase LC columns, they employed HILIC column [23]. This approach demonstrates the versatility of LC‐MS/MS coupled with the strategic use of different chromatographic columns to effectively analyse a diverse range of drugs. As described by Dugheri et al. [15], the method employed is a two run‐time LC‐MS/MS analysis, where the sample undergoes two separate runs: one in ESI+ and another in ESI−. This strategy ensures the detection of a wide range of antineoplastic drugs, with each mode tailored to the specific properties of the compounds being analysed.

The analysed molecules exhibited diverse physicochemical properties, making their simultaneous quantification challenging. However, HILIC chromatography, known for its ability to retain polar compounds, emerged as the preferred method due to its capability to retain and separate these diverse molecules effectively. By using an HILIC phase, it became possible to achieve the simultaneous quantification of 5‐FU with several other molecules, such as cyclophosphamide, doxorubicin and methotrexate, within a short time frame of 8 min. In comparison, alternative methods described in literature surpassed this time limit significantly with Dugheri et al. [24] reporting a method duration of 23 min and Fleury et al. [25] documenting a method duration of 13 min.

Indeed, various authors have recommended monitoring 5‐FU, but they typically propose two methods with different chromatographic support, detection and chromatographic parameters or different analytical methods [5, 8, 26].

Rossignol et al. [27] proposed an HPLC/MS‐MS method using C18 stationary phase, but our method demonstrated a superior LOQ for 5‐FU, doxorubicin and epirubicin (0.01, 0.02 and 0.02 ng/cm^2^ compared to 0.025 ng/cm^2^). Jeronimo et al. [9] proposed an LC‐MS/MS method using a biphenyl column but with higher LOQs for 5‐FU (1 ng/mL compared to 15.55 ng/mL). Nussbaumer et al. [3] proposed an HPLC/MS‐MS method using C18 stationary phase, but our method demonstrated a superior LOQ for gemcitabine and irinotecan and a comparable one for doxorubicin and epirubicin (0.4, 0.4, 0.2 and 0.2 ng/mL, respectively, compared to 0.5, 1, 0.2 and 0.2 ng/mL).

One advantage of employing HILIC conditions over reverse‐phase HPLC is the ability to use a high proportion of ACN, which enhances ionization efficiency for mass spectrometry (MS) detection [28].

Ares and Bernal [11] demonstrated the effectiveness of HILIC chromatography for the simultaneous determination of both polar and non‐polar cytotoxic drugs. Their findings support our approach, particularly in highlighting the ability of HILIC to retain highly polar compounds such as 5‐FU while maintaining high resolution for less polar analytes.

### 3.2 Method Validation

The results of method validation are summarized in Data S3. Calibration curves were constructed within a concentration range from the LOQ to 200 ng/mL as detailed in Data S3, using standard solutions at concentration of 4000, 400 and 40 ng/mL.

The calibration curve demonstrated linearity for doxorubicin and epirubicin, while a polynomial (*x* ^2^) behaviour was observed for the other molecules. The determination coefficient (*r* ^2^) was 0.98 for 5‐FU and exceeded 0.99 for the other molecules. The RSD was less than 5% for intra‐day precision (repeatability) and less than 15% for inter‐day precision (intermediate precision). These results confirm the reliability of the method for routine monitoring of antineoplastic drug contamination. The trueness percentage ranged within ±20%, which is considered satisfactory for the quantitative determination of residual contaminations.

The LOQ values were expressed in ng/mL and ng/cm^2^ to facilitate comparison with other studies. All LOQ were less than 1 ng/cm^2^, aligning with the established limit for residual contamination of cyclophosphamide, ifosfamide, 5‐FU and methotrexate as set by the USP [16].

Our method provided LOQ values comparable to those reported in other studies. The LOQs were 0.01, 0.04, 0.01, 0.004, 0.02, 0.02, 0.04 and 0.004 ng/cm^2^ for 5‐FU, ifosfamide, cyclophosphamide, gemcitabine, doxorubicin, epirubicin, methotrexate and irinotecan, respectively_._

The method was validated according to international guidelines (ICH), confirming its robustness for the intended application. The selection of MS parameters, including ionization mode, collision energy and mass transitions, was guided by preliminary experiments to optimize signal intensity and minimize matrix effects.

### 3.3 Method Application

The application of the method revealed that among the 28 wiped surfaces, 22 surfaces were contaminated by at least one of the eight specified anticancer drugs. Detailed results can be found in Data S4.

Ifosfamide was detected on 14 surfaces, while 5‐FU was found on 10 surfaces. Notably, 5‐FU is the most commonly used antineoplastic drug in our hospital and is often prescribed in high doses. Residual contamination with at least one of the eight tested antineoplastic drugs was observed on all surfaces wiped in the patient's toilet. The highest level of contamination was attributed to ifosfamide, with a maximum contamination of 0.66 ng/cm^2^ detected on one of the wiped surfaces in the patient's toilet.

Many studies, including those conducted by Dugheri et al. and Fleury et al., have shown that the antineoplastic drugs handled in the highest quantities, both in terms of milligrams and number of vials, tend to produce the most contamination [24, 25].

Residual contamination with ifosfamide was found on 14 surfaces, with the highest average of contamination level recorded at 0.19 ± 0.24 ng/cm^2^, despite the low use of this molecule in our hospital (less than 1% of preparations). This finding suggests the possibility of an inappropriate cleaning procedure for this molecule [7, 29]. In fact, several authors pointed out challenges in cleaning efficacy, explaining residual contamination of some surfaces [30, 31].

The presence of residual contamination of antineoplastic in the patient rooms, even after the cleaning, suggests that the cleaning procedures may be insufficient or inadequately implemented. Ifosfamide and 5‐FU were the most frequently detected molecules, with the highest mean contamination levels. These two molecules are commonly identified in surface contamination monitoring efforts [6, 7, 8, 29, 32] underscoring the importance of including them in our study.

Actually, there is no consensus on the tolerable residual contamination thresholds and for that results interpretation remains controversial; as USP suggests a limit of 1 ng/cm^2^, the European Biosafety Network has recommended a lower limit value of 100 pg/cm^2^, the same as in Kiffmeyer et al. [33, 34]. In our study, some surfaces exhibited residual contamination exceeding this threshold (patient's toilet for doxorubicin and ifosfamide, and nursing station for 5‐FU and gemcitabine).

Similarly, in the 2022 study [24], 5‐FU, gemcitabine, cyclophosphamide and ifosfamide showed notable detection rates. The same goes for the Kiffmeyer et al.’s [33] study for cyclophosphamide, gemcitabine and 5‐FU, and the Bláhová et al.’s [35] study for platinum‐based drugs, cyclophosphamide, gemcitabine and 5‐FU.

The widespread detection of antineoplastic drugs on hospital surfaces reflects the limitations of current cleaning protocols. For instance, cyclophosphamide was detected on 75% of sampled surfaces, indicating persistent contamination despite routine cleaning. This finding highlights the need for enhanced decontamination strategies, particularly in high‐risk areas such as drug preparation rooms and patient care units.

The method also enabled the detection of unknown molecules, one of which was identified as a detergent residue. This underscores the versatility of the developed method, which not only quantifies antineoplastic drugs but also identifies other surface contaminants that may interfere with cleaning efficacy or pose additional risks.

#### 3.3.1 Screening of Untargeted Molecules

**FIGURE 3:**
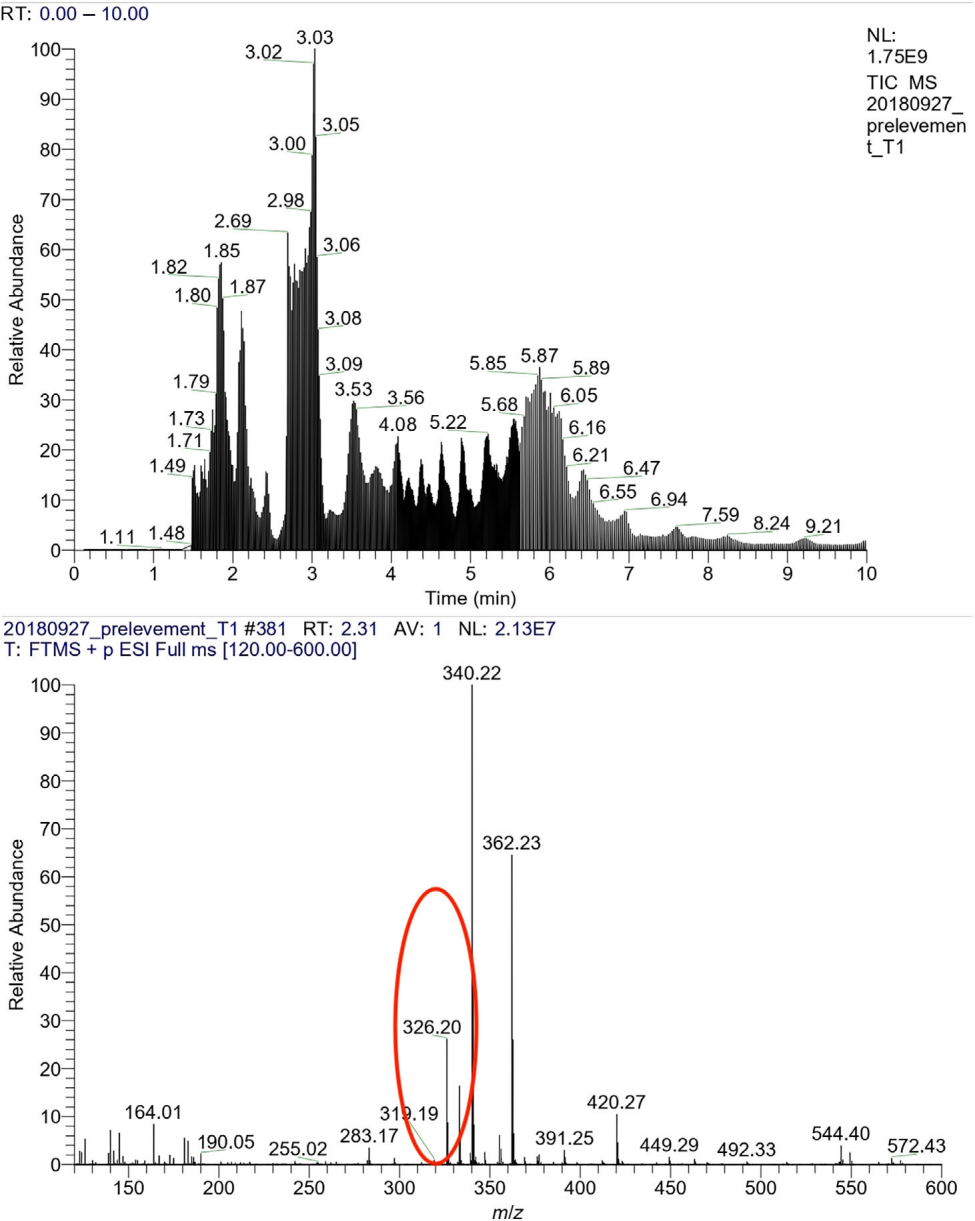
TIC of a contaminated surface with antineoplastics and detergent.

During the screening of untargeted molecules in the analysed samples, the presence of various molecules, in addition to those of interest, was noted. A specific ion, detecting in various wipes, was found to be consistent with the surface‐cleaning product. This ion, with a detected mass of *m/z* 326.2 at the same retention time was identified as dodecyl dimethyl ammonium chloride, a compound found in the detergent used for surface cleaning in our hospital, based on its mass spectra studied by Núñez et al. [36], and the parent ion was *m/z* 326.2 as shown in Figure 3.

Several other ions were also detected but attempts to identify them using published spectral libraries did not yield relevant information. This suggests that surface contamination could involve not only cytotoxic drugs but also degradation products, as the surfaces are usually exposed to mechanical stress, UV radiation and temperature. Therefore, the information obtained from surface wiping should be further investigated.

It would be beneficial to identify ions associated with specific ‘retention time, *m/z*’ pairs found across multiple surfaces. This approach would allow us to focus on the most intense signals and frequently detect ions provided by the high‐resolution detector. Such ions could potentially represent degradation products of antineoplastic drugs whose toxicity for human is not well‐known.

Forced degradation of antineoplastic drugs could be conducted on clean surfaces to generate potential degradation products. Subsequently, the retention times and spectra of these products could be compared with those found on surfaces.

Residual contamination of surfaces with anticancer drugs has been established by multiple studies [8, 18, 27, 37–40]. However, the presence of degradation products should also be considered. The high specificity of the high‐resolution mass spectrometry (HR‐MS/MS) detector allows for accurate characterization of the working environment, as demonstrated by Dal Bello et al. [5], and could be applied for monitoring antineoplastic metabolites and degradation products in complex matrices such as wastewater [5].

#### 3.3.2 Comparison With Previous Works

The contamination levels observed in this study are consistent with those in previous research but highlight significant variability depending on the hospital unit and cleaning protocol. This underscores the importance of standardized monitoring methods, such as the one developed here, to ensure reliable comparisons across different facilities. The results demonstrate the effectiveness of the developed HILIC‐MS/MS methodology for the simultaneous detection of multiple antineoplastic drugs on hospital surfaces. Compared to previous studies, such as those by Rossignol et al. [27], our method offers superior sensitivity and the ability to detect drugs at lower concentrations. The surface wipe sampling technique used in this study provides a more comprehensive assessment of contamination patterns across various hospital zones, contributing valuable data for occupational health.

Our HILIC‐MS/MS method was compared to several previously published methods, particularly those by Dugheri et al., who explored the use of zwitterionic HILIC for the determination of platinum‐based and broader classes of antineoplastic drugs on surfaces [14, 15]. While these studies provided valuable insights into the use of HILIC for surface contamination monitoring, our method offers significant improvements in terms of sensitivity and the ability to detect a broader range of drugs.

Dugheri et al. [14] focused primarily on platinum‐based drugs, while Dugheri et al. [15] expanded their work to include other classes of antineoplastic agents. In contrast, our study uses a non‐zwitterionic HILIC approach, which allows for the simultaneous detection of a wider spectrum of antineoplastic drugs, including both alkylating agents and mitotic inhibitors, which are often difficult to detect with other chromatographic methods such as RPLC due to their polarity and low volatility. In this context, while Orbitrap MS provides high resolution and sensitivity, the detection of extremely low concentrations can still present challenges. Achieving reliable detection at these levels requires optimized conditions, such as improved ionization and ion accumulation, as low concentrations may not generate sufficient signal without fine‐tuning the instrument parameters. Our method incorporates high‐resolution Orbitrap MS, which provides a more comprehensive detection capability by enabling the identification of non‐targeted compounds, such as degradation products and cleaning agents, which have not been systematically addressed in previous studies. These improvements make our method more suitable for environmental contamination monitoring in healthcare settings.

## 4 Conclusion

Our study enabled the simultaneous determination of residues of several commonly used antineoplastic molecules on surfaces using a rapid, robust HILIC‐MS/MS method. This method, validated for quantifying eight intravenous antineoplastic drugs, demonstrated excellent sensitivity and specificity, with quantification limits below 0.04 ng/cm^2^. The optimization of experimental parameters, including mobile phase composition and flow rate, further enhanced its performance. The inclusion of high‐resolution Orbitrap MS significantly expanded the method's capabilities, enabling the identification of both targeted and non‐targeted compounds, such as degradation products, premedication drugs and cleaning agents.

Application of this method to real‐world hospital surfaces revealed widespread contamination, with 22 of 28 samples testing positive for at least one drug. The detection of unknown compounds highlights the potential for further investigations into contamination sources and the efficacy of cleaning protocols. By addressing the challenge of analysing a chemically diverse group of molecules using a single protocol, this method represents a significant advancement in analytical sciences, surpassing traditional techniques like RPLC, which often struggle with polar compounds.

This novel analytical technique offers a rapid, high‐sensitivity approach for effective monitoring of surface contamination, contributing to enhanced safety standards and improved hospital hygiene practices. Its adaptability for detecting a broad range of contaminants, not only antineoplastic drugs but also associated compounds, within a short analysis time, its sensitivity and its potential for automation make it a valuable tool for routine monitoring and addressing occupational exposure risks. Further research will focus on expanding the range of detectable molecules, identifying additional unknown contaminants and exploring its applicability in other matrices, such as air or personal protective equipment. Ultimately, this research provides a strong foundation for improving healthcare worker safety, advancing contamination control and enhancing regulatory standards for handling hazardous drugs in healthcare settings.

## Author Contributions

**Zribi Kaouther**: conceptualization, analysis, and writing; **Sarra Berriri**: analysis and writing; **Danielle Libong**: review; **Audrey Solgadi**: analysis; **Fathi Safta**: conceptualization; **Laetitia Minh Mai Lê**: conceptualization and analysis; **Eric Caudron**: conceptualization and review.

## Conflicts of Interest

The authors declare no conflicts of interest.

## Supporting information



Supporting Information

## Data Availability

The authors have nothing to report.
