# Supplementary material for: Simultaneous Determination of Residual Contamination of Eight Antineoplastic on Surfaces by HILIC Chromatography Coupled to High‐Resolution Spectrometry
Source: Anal Sci Adv. 2025 Feb 27;6(1):e70004. doi: 10.1002/ansa.70004 (PMC11867787; doi:10.1002/ansa.70004)
Supplement: Supplementary file 1 — Supporting Information [file ANSA-6-e70004-s001.docx]

**SUPPLEMENTARY MATERIAL**

**Supplementary data 1: Applied linear gradient**

| Time (min) | % of water with 0.1% acid formic | % of ACN with 0.1% acid formic |
| --- | --- | --- |
| 0 - 1 | 5 | 95 |
| 1 - 1,5 | 15 | 85 |
| 1,5 - 7 | 15 | 85 |
| 7 - 7,5 | 5 | 95 |
| 7,5 - 20 | 5 | 95 |

**Supplementary data 2 : HPLC-HR-MS/MS conditions for the analyzed antineoplastic drugs**

|  | Precursor ion m/z | Quantitative product ion m/z | Collision Energy (eV) | Retention time (min) |
| --- | --- | --- | --- | --- |
| 5 Fluorouracil | 129.0  [M-H^+^]^-^ | NA | 30 | 1.26 |
| Ifosfamide | 261.1  [M+H^+^]^+^ | 182,0 | 25 | 1.76 |
| Cyclophosphamide | 261.1  [M+H^+^]^+^ | 140,0 | 25 | 1.86 |
| Gemcitabine | 264.3  [M+H^+^]^+^ | 112,1 | 20 | 4.63 |
| Doxorubicin | 544.3  [M+H^+^]^+^ | 397,1 | 15 | 5.76 |
| Epirubicin | 544.4  [M+H^+^]^+^ | 397,1 | 15 | 5.88 |
| Methotrexate | 455.4  [M+H^+^]^+^ | 308,2 | 30 | 5.82 |
| Irinotecan | 587.5  [M+H^+^]^+^ | 543,3 | 35 | 6.72 |

**Supplementary data 3: Validation parameters**

|  | 5 FU | Ifosfamide | Cyclophosphamide | Gemcitabine | Doxorubicin | Epirubicin | Methotrexate | Irinotecan |
| --- | --- | --- | --- | --- | --- | --- | --- | --- |
| Trueness (%) | 84.5-120 | 80-111 | 89-111 | 83-117 | 84-120 | 83-107 | 92-119 | 89-113 |
| Repeatability (%) | 1.5-16 | 0.94-4.6 | 0,3-4 | 0.2-1.4 | 0-2.7 | 0.9-6 | 0.1-2.9 | 0.1-1.6 |
| Intermediate precision (%) | 2-12 | 2.6-5.2 | 1-4 | 0,7-16 | 2,5-12 | 0,4-9 | 1,7-8,3 | 0.2-11 |
| Limit of quantification (ng/mL) | 1 | 4 | 1 | 0.4 | 2 | 2 | 4 | 0.4 |
| Limit of quantification (ng/cm^2^) | 0.01 | 0.04 | 0.01 | 0.004 | 0.02 | 0.02 | 0.04 | 0.004 |
| Linearity range (ng/mL) | 1-100 | 4-200 | 1-200 | 0,4-200 | 2-200 | 2-200 | 4-200 | 0,4-200 |
| Correlation coefficient | 0.9892  (polynomial (x2)) | 0,9989  (polynomial (x2)) | 0,9985  (polynomial (x2)) | 0,9995  (polynomial (x2)) | 0,9988 (linear) | 0,9991 (linear) | 0,9998  (polynomial (x2)) | 0,9999  (polynomial (x2)) |

**Supplementary data 4 : List of contaminated samples for each molecule and average of contamination (ng.cm^-2^)**

LD : Limit of Detection

|  |  | 5-FU | Ifosfamide | Cyclophosphamide | Gemcitabine | Doxorubicin | Methotrexate | Epirubicin | Irinotecan |
| --- | --- | --- | --- | --- | --- | --- | --- | --- | --- |
| Patient's room  (4 contaminated samples /6) | Number of contaminated samples | 0 | 4 | 0 | 0 | 0 | 0 | 0 | 3 |
|  | Average of contamination (ng.cm^-2^) | < LD | 0.061 | < LD | < LD | < LD | < LD | < LD | 0.02 |
| Patient'stoilet  (6 contaminated samples/6) | Number of contaminated samples | 3 | 3 | 1 | 0 | 1 | 0 | 0 | 3 |
|  | Average of contamination(ng.cm^-2^) | 0.07 | 0.46 | 0.02 | < LD | 0.14 | < LD | < LD | 0.02 |
| Nursing station  (12 contaminated samples/16) | Number of contaminated samples | 7 | 7 | 2 | 4 | 0 | 2 | 0 | 0 |
|  | Average of contamination(ng.cm^-2^) | 0 .14 | 0.06 | 0.04 | 0.10 | < LD | 0.08 | < LD | < LD |
| Total | Contaminated samples | 10 | 14 | 3 | 4 | 1 | 2 | 0 | 6 |
|  | Average of contamination(ng.cm^-2^) | 0.127  ±0.20 | 0.194  ±0.24 | 0.032  ±0.02 | 0.10  ±0.12 | < LD | 0.075 | < LD | 0.019  ±0.006 |
|  | Residual contamination  > 0,1ng.cm^-2^ | 4 | 4 | 0 | 1 | 1 | 0 | 0 | 0 |
|  | Residual contamination  > 1ng.cm^-2^ | 0 | 0 | 0 | 0 | 0 | 0 | 0 | 0 |
